# Maize phenylalanine ammonia‐lyases contribute to resistance to *Sugarcane mosaic virus* infection, most likely through positive regulation of salicylic acid accumulation

**DOI:** 10.1111/mpp.12817

**Published:** 2019-09-05

**Authors:** Wen Yuan, Tong Jiang, Kaitong Du, Hui Chen, Yanyong Cao, Jipeng Xie, Mengfei Li, John P. Carr, Boming Wu, Zaifeng Fan, Tao Zhou

**Affiliations:** ^1^ State Key Laboratory for Agro‐Biotechnology China Agricultural University Beijing 100193 China; ^2^ Ministry of Agriculture and Rural Affairs, Key Laboratory for Pest Monitoring and Green Management, Department of Plant Pathology China Agricultural University Beijing 100193 China; ^3^ Cereal Crops Institute Henan Academy of Agricultural Science Zhengzhou 450002 China; ^4^ Department of Plant Sciences University of Cambridge Downing Street Cambridge CB2 3EA UK

**Keywords:** ligin, phenylalanine ammonia‐lyase, phenylpropanoid biosynthesis, resistance, salicylic acid, *Sugarcane mosaic virus*, virus‐induced gene silencing

## Abstract

*Sugarcane mosaic virus* (SCMV) is a pathogen of worldwide importance that causes dwarf mosaic disease on maize (*Zea mays*). Until now, few maize genes/proteins have been shown to be involved in resistance to SCMV. In this study, we characterized the role of maize phenylalanine ammonia‐lyases (ZmPALs) in accumulation of the defence signal salicylic acid (SA) and in resistance to virus infection. SCMV infection significantly increased SA accumulation and expression of SA‐responsive pathogenesis‐related protein genes (*PR*s). Interestingly, exogenous SA treatment decreased SCMV accumulation and enhanced resistance. Both reverse transcription‐coupled quantitative PCR and RNA‐Seq data confirmed that expression levels of at least four *ZmPAL* genes were significantly up‐regulated upon SCMV infection. Knockdown of *ZmPAL* expression led to enhanced SCMV infection symptom severity and virus multiplication, and simultaneously resulted in decreased SA accumulation and *PR* gene expression. Intriguingly, application of exogenous SA to SCMV‐infected *ZmPAL*‐silenced maize plants decreased SCMV accumulation, showing that ZmPALs are required for SA‐mediated resistance to SCMV infection. In addition, lignin measurements and metabolomic analysis showed that ZmPALs are also involved in SCMV‐induced lignin accumulation and synthesis of other secondary metabolites via the phenylpropanoid pathway. In summary, our results indicate that ZmPALs are required for SA accumulation in maize and are involved in resistance to virus infection by limiting virus accumulation and moderating symptom severity.

## Introduction

Maize (*Zea mays*) is the second largest crop by area harvested in the world (FAOSTAT, 2017). In many regions, especially in Europe and China, maize production is threatened by *Sugarcane mosaic virus* (SCMV) (Shukla *et al*., 1992). SCMV, a member of genus *Potyvirus*, causes significant losses in production of maize, sugarcane, sorghum and many other monocotyledonous species (Fuchs and Gruntzig, 1995; Shi *et al*., 2006). Annually, SCMV infection causes losses of 10–50% of the maize yield in China, with the Beijing isolate (SCMV‐BJ) as the prevalent strain (Chiu, 1988; Fan *et al*., 2003; Jiang and Zhou, 2002; Li *et al*., 2013).

Chemical and agronomic methods are used to control SCMV. For example, killing aphid vectors with pesticides is widely used to attempt to prevent SCMV infection. However, frequent application of pesticides raises serious food safety and environmental concerns and, in any case, insecticides do not provide effective control of non‐persistently transmitted viruses (Groen *et al*., 2017). Several maize cultivars carrying SCMV resistance loci (i.e. *ZmTrxh* and *ZmABP1*) are in commercial production (Leng *et al*., 2017; Liu *et al*., 2017; Tao *et al*., 2013; Xu *et al*., 1999), but newer, more virulent SCMV isolates have been observed recently in maize fields (Gao *et al*., 2011; Yan *et al*., 2016). Consequently, it is important to develop improved SCMV control strategies, including production of genetically modified or gene‐edited maize cultivars or the use of resistance‐inducing chemicals.

Substantial efforts have been devoted to understanding the molecular interactions between maize and SCMV in order to develop sustainable maize protection strategies. For instance, several maize proteins have been identified that interact directly with SCMV‐encoded proteins (Chen *et al*., 2017b; Cheng *et al*., 2008; Zhu *et al*., 2014). Gene and protein expression profiling have also been conducted to elucidate the response of maize to SCMV infection (Chen *et al*., 2017a; Shi *et al*., 2005, 2006; Uzarowska *et al*., 2009; Wu *et al*., 2013a). However, until now, only two maize proteins (polyamine oxidase and violaxanthin deepoxidase protein) have been shown to improve resistance to SCMV (Chen *et al*., 2017a, 2017b). Thus, further studies of maize resistance to SCMV are required.

It is well known that the phytohormone salicylic acid (SA; 2‐hydroxybenzoic acid) plays a critical role in plant development, as well as in defence against many pathogens and abiotic stresses (Vicente and Plasencia, 2011). However, the biosynthetic pathway for SA has been determined in only a few plant species. In *Arabidopsis thaliana* and barley (*Hordeum vulgare*), most SA biosynthesis is dependent on isochorismate synthase (ICS) activity (Hao *et al*., 2018; Wildermuth *et al*., 2001). In tobacco (*Nicotiana tabacum*) SA is predominantly synthesized from benzoic acid derived from the phenylpropanoid pathway (phenylalanine) (Lee *et al*., 1995), in peach (*Prunus persica*) SA is also produced from benzoic acid derived from phenylalanine via an alternative pathway (Diaz‐Vivancos *et al*., 2017), whilst in soybean (*Glycine max*) the isochorismate and phenylpropanoid pathways are equally important in supplying the carbon skeletons for SA biosynthesis (Shine *et al*., 2016). The phenylpropanoid pathway is also involved in the biosynthesis of many plant defensive compounds, including flavonoids, lignin, condensed tannins, hydroxycinnamic acid, coumarins and stilbenes (An and Mou, 2011; Yu and Jez, 2008). Though SCMV infection causes SA accumulation in susceptible and resistant inbred lines of maize (Wu *et al*., 2013b), the biosynthetic pathway and its role in defence against virus infection in maize remain unclear.

Phenylalanine ammonia‐lyase (PAL) catalyses deamination of l‐phenylalanine to *trans*‐cinnamate and is the key enzyme regulating carbon flux through the phenylpropanoid pathway (Dixon and Paiva, 1995; Hahlbrock and Scheel, 1989; Huang *et al*., 2010; Vogt, 2010). *Arabidopsis*, soybean and pepper (*Capsicum annuum*) have multiple *PAL* genes (Huang *et al*., 2010; Kim and Hwang, 2014; Shine *et al*., 2016). *PAL*s are responsive to pathogen infection, nutrient stress, wounding, UV and elevated temperature (Dixon and Paiva, 1995; Edwards *et al*., 1985; Huang *et al*., 2010; Jin *et al*., 2013; Kim and Hwang, 2014; Liang *et al*., 1989; MacDonald and D’Cunha, 2007; Payyavula *et al*., 2012). PAL activity is reported to influence SA accumulation in *Arabidopsis*, tobacco, pepper and soybean (Kim and Hwang, 2014; Mauch‐Mani and Slusarenko, 1996; Pallas *et al*., 1996; Shine *et al*., 2016). Through disruption of *PAL* gene expression or PAL enzyme activity, the roles of PAL in plant development and responses to external stimuli have been illustrated in several plant species (Huang *et al*., 2010; Kim and Huang, 2014; Pallas *et al*., 1996; Rohde *et al*., 2004; Shine *et al*., 2016). For example, inoculation with tobacco mosaic virus (TMV) of *NN* genotype (TMV‐resistant) tobacco plants induces SA accumulation, which is associated with containment of the virus to the infection site (the hypersensitive response) and establishment of enhanced resistance (systemic acquired resistance; SAR) to subsequent infections (Malamy *et al*., 1990; Yalpani *et al*., 1991). SA accumulation was diminished and SAR was inhibited in transgenic plants in which *PAL* gene expression was suppressed (Felton *et al*., 1999; Pallas *et al*., 1996). Application of the PAL inhibitor 2‐aminoindan‐2‐phosphonic acid to potato, cucumber or *Arabidopsis* plants inhibited accumulation of pathogen‐ or elicitor‐induced SA (Coquoz *et al*., 1998; Mauch‐Mani and Slusarenko, 1996; Meuwly *et al*., 1995). *Arabidopsis* plants with mutations in the four *AtPAL* genes showed increased susceptibility to *Pseudomonas syringae* infection accompanied by decreased levels of basal and pathogen‐induced SA and lignin (Huang *et al*., 2010). Pepper plants silenced for expression of *CaPAL1* were more susceptible to *Xanthomonas campestris* pv. *vesicatoria* and had decreased levels of SA and *CaPR1* gene expression (Kim and Huang, 2014). In rice, *OsPAL6*‐knockout mutant plants were highly susceptible to *Magnaporthe oryzae* and susceptibility was associated with decreased flavonoid, phytoalexin and SA accumulation (Duan *et al*., 2014). Knockdown of *PAL* expression in soybean inhibits SA biosynthesis and resistance to *P. syringae* and *Phytophthora sojae* (Shine *et al*., 2016; Zhang *et al*., 2017).

Maize has ten putative *PAL* genes, listed as *ZmPAL* and *ZmPALs 1–9* in the updated maize genome database (AGPv4, http://ensembl.gramene.org/Zea_mays/Info/Index). However, no detailed analysis or functional studies have been reported for them. Only two maize *PAL* genes (originally named *ZmPAL1* and *ZmPAL2*) have been cloned. Both were confirmed to encode proteins with PAL and tyrosine ammonia‐lyase (TAL) activities that effectively catalysed conversion of l‐phenylalanine to *trans*‐cinnamic acid (Rosler *et al*., 1997; Zang *et al*., 2015). It was shown that the expression of four *ZmPAL* genes (originally named *ZmPAL1*, *ZmPAL2*, *ZmPAL4* and *ZmPAL5*) was strongly induced after nematode infection, while two others (*ZmPAL3* and *ZmPAL6*) were unresponsive (Starr *et al*., 2014). To date, the phylogenetic relationships among *ZmPAL* genes and their roles in maize defence against pathogen infections are unclear.

In this study, we determined that SCMV infection induced SA biosynthesis, enhanced expression of *ZmPAL* genes and the accumulation of secondary metabolites derived from the phenylpropanoid pathway. Knockdown of expression of *ZmPAL* genes decreased SA accumulation and exacerbated SMCV infection. Interestingly, SCMV‐induced accumulation of endogenous SA appears to moderate the accumulation of the virus.

## Results

### Exogenous SA treatment inhibits SCMV infection in maize

**Figure 1:**
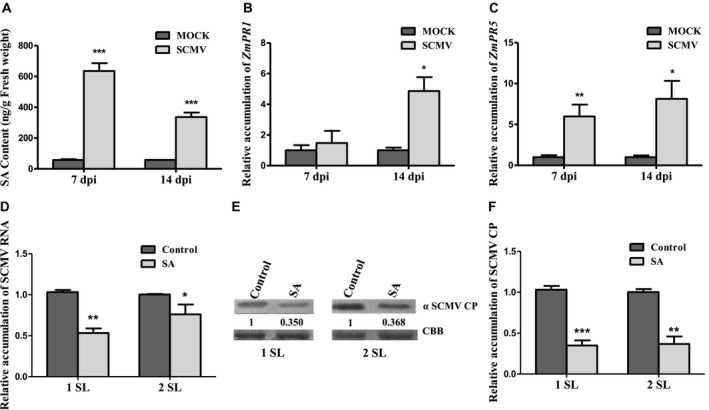
Salicylic acid (SA) contributes to maize defence against *Sugarcane mosaic virus* (SCMV) infection. (A) Concentrations of SA in maize (*Z. mays* inbred line B73) plants treated with phosphate buffer (mock) or infected with SCMV were measured at 7 and 14 days post‐inoculation (dpi), respectively. (B) and (C) Expression levels of pathogenesis‐related protein genes *PR1* and *PR5* were measured by RT‐qPCR in systemically infected maize leaves or equivalent leaves from mock‐inoculated plants at 7 and 14 dpi, respectively. (D) Relative accumulation of SCMV RNA in systemically infected leaves of the SA treated or control (sterile distilled water (H_2_O)‐treated) plants. (E) Accumulation of SCMV CP in systemically infected leaves of SA treatment or control maize measured by western blotting and visualized by ImageJ software. The numbers shown below the upper panel indicated the ratio of SCMV CP accumulation in the SA treatment or control maize leaves. The relative accumulation of SCMV CP in the plants treated with the control solution [0.2% (v/v) Tween‐20: labelled H_2_O) maize plants is presented as 1. (F) Relative accumulation of SCMV CP in systemically infected leaves of SA treatment or control maize measured by ImageJ software. Three independent experiments were conducted with at least three biological replicates per treatment. Error bars represent the mean ± SE. Statistical significances are indicated (*P < 0.05, **P < 0.01, ***P < 0.001) and were determined using Student’s *t*‐test. 1 SL, the first systemically infected leaf; 2 SL, the second systemically infected leaf.

Systemic infection of susceptible plants by certain compatible viruses, for example during the infection of *nn* genotype tobacco by TMV, does not result in increased SA biosynthesis by the host (Malamy *et al*., 1990). However, cauliflower mosaic virus (CaMV) (Love *et al*., 2005), cucumber mosaic virus (Zhou *et al*., 2014), potato virus Y (Krečič‐Stres *et al*., 2005) and turnip mosaic virus (Whitham *et al*., 2003) induce increased SA accumulation and SA‐responsive gene expression as they spread through their susceptible hosts. SCMV infection was previously reported to cause SA accumulation in maize inbred lines (Wu *et al*., 2013b), but the role of SA in SCMV–maize interactions was not clarified. In this study, we first determined that SA levels in the SCMV‐infected plants increased by 10.1‐ and 3.8‐fold at 7 and 14 days post‐inoculation (dpi), respectively, compared with levels in mock‐inoculated plants (Fig. 1A). Consistent with this, steady‐state transcript levels for the SA‐responsive marker genes *ZmPR1* up‐regulated by 3.9‐fold at 14 dpi, and *ZmPR5* up‐regulated by 5.2‐ and 7.4‐fold at 7 and 14 dpi, respectively (Fig. 1B,C).

Although certain viruses induce SA biosynthesis in their susceptible hosts, the accumulation of these viruses is nevertheless inhibited if host plants are pre‐treated with exogenous SA or if endogenous SA biosynthesis is triggered prior to infection by an incompatible pathogen (Ji and Ding, 2001; Mayers *et al*., 2005; Naylor *et al*., 1998). To determine if treatment with exogenous SA induces resistance to SCMV infection in maize, SA was applied to maize plants prior to virus inoculation. At the concentration used SA did not inhibit plant growth (Fig. S1) but it did decrease susceptibility to the virus (Fig. 1D–F). The accumulation of SCMV RNA and coat protein (CP) in the infected plants was measured by reverse transcription‐coupled quantitative PCR (RT‐qPCR) and western blot analyses, respectively (Fig. 1D–F). Compared with control‐treated plants, the accumulation of SCMV genomic RNA was decreased by almost half in the first systemically infected leaves (1 SL) and approximate 25% in the second systemically infected leaves (2 SL) of the SA‐treated plants (Fig. 1D). SCMV CP accumulation was also significantly decreased in SA‐treated plants (Fig. 1E,F). Thus although SA accumulation is triggered in maize by systemic infection with SCMV, infection by this virus is inhibited by SA when the chemical is applied prior to infection (Fig. 1).

### SCMV infection induces expression of *ZmPAL* genes

To test whether maize PALs were involved in SA‐mediated resistance to SCMV, we first analysed the phylogeny of *ZmPAL* genes and then investigated their expression patterns after SCMV infection.

Ten maize *PAL* gene sequences were obtained from the updated *Z. mays* B73 genome (AGPv4, http://ensembl.gramene.org/Zea_mays/Info/Index): *ZmPAL* (Zm00001d017274), *ZmPAL1* (Zm00001d029015), *ZmPAL2* (Zm00001d003016), *ZmPAL3* (Zm00001d051161), *ZmPAL4* (Zm00001d051166), *ZmPAL5* (Zm00001d051163), *ZmPAL6* (Zm00001d003015), *ZmPAL7* (Zm00001d017279), *ZmPAL8* (Zm00001d017276) and *ZmPAL9* (Zm00001d017275) (Fig. S2). Sequence alignments indicated that these ten *PAL* genes shared about 45% nucleotide sequence identity and about 80% amino acid sequence identity (Figs S2 and S3). Phylogenetic analysis using deduced amino acid sequences indicated that the ten *ZmPAL* genes could be divided into two clades. *ZmPAL*, *ZmPAL2* and *ZmPAL3* are in Clade II and other seven PALs belong to Clade I (Fig. S4).

The deduced amino acid sequences of ZmPALs were aligned with the PALs of *Arabidopsis*, tobacco, tomato (*Solanum lycopersicum*), potato (*S. tuberosum*), pepper, *Ipomoea* and rice. Amino acid sequence identities ranged from 11.5% to 99.4% (Fig. S5). Phylogenetic analysis showed that *PAL* genes of monocotyledonous species clustered separately from those of dicotyledonous species (Fig. S6).

**Figure 2:**
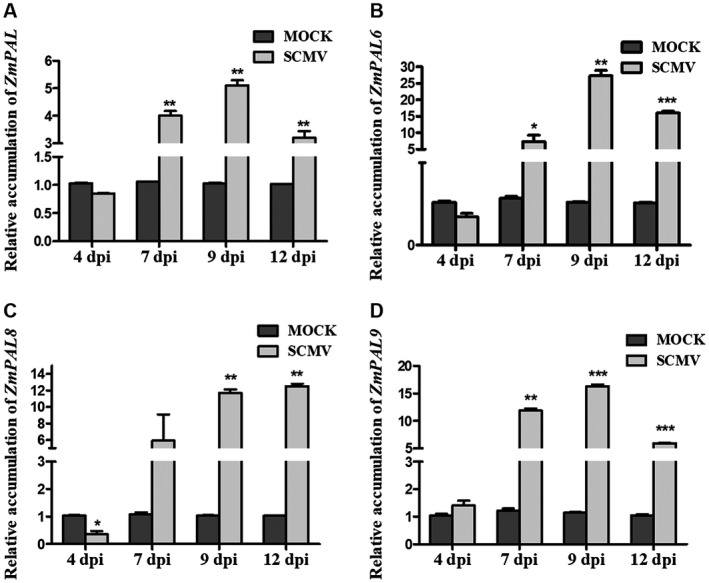
SCMV infection regulates expression of *PAL* genes in maize. (A)–(D). Relative expression levels of *ZmPAL*, *ZmPAL6*, *ZmPAL8* and *ZmPAL9* transcripts in SCMV‐infected or mock‐inoculated plants. Three independent experiments were conducted with at least three biological replicates per treatment. Error bars represent the mean ± SE. Statistically significant differences are indicated (*P < 0.05, **P < 0.01, ***P < 0.001) and were determined using Student’s *t*‐test.

We used RT‐qPCR to determine relative expression of different *ZmPAL* transcripts in mock‐inoculated and SCMV‐infected maize plants (Fig. 2). At 7 dpi, the steady‐state accumulation of the *ZmPAL* (Zm00001d017274) transcript in systemically infected leaves was about 3‐fold higher than that in the equivalent leaves of mock‐inoculated plants (Fig. 2A). By 9 dpi, *ZmPAL* gene expression in systemically infected leaves was about 5‐fold greater than in the equivalent leaves of mock‐inoculated plants but had declined by 12 dpi (Fig. 2A). Expression of *ZmPAL6*, *ZmPAL8* and *ZmPAL9* was also strongly induced by SCMV infection at 7, 9 and 12 dpi (Fig. 2D–F). Of the remaining six *ZmPAL* genes, we could not detect increases in expression by RT‐qPCR because of the lack of gene‐specific primers. Since RT‐qPCR analysis (Fig. 2) indicated that the greatest induction of *ZmPAL* gene expression had occurred by 9 dpi, we performed RNA‐Seq on SCMV‐infected and mock‐inoculated maize tissues harvested at this time point. This analysis confirmed that expression of *ZmPAL* and *ZmPALs 4, 6*, *7*, *8* and *9* was increased significantly after 9 days of SCMV infection. The expression of *ZmPAL2*, *ZmPAL3* and *ZmPAL5* was not affected by SCMV infection (Fig. S7). Consistent with the RT‐qPCR data, no *ZmPAL1* RNA transcript was detected in maize by RNA‐Seq, indicating that *ZmPAL1* may not be expressed. Taking the results of the phylogenetic and expression analyses together, there appears to be no relationship between *ZmPAL* genes belonging to Clade I or II (Fig. S4) and their ability to be induced by viral infection (Figs 2 and S7).

### Knockdown of  *ZmPAL* gene expression enhances SCMV accumulation and symptom severity, and decreases SA accumulation

We used the *Brome mosaic virus* (BMV)‐derived virus‐induced gene silencing (VIGS) vector to knockdown *ZmPAL*  transcript accumulation. BMV‐VIGS has been used successfully to decrease expression of specific maize genes for interaction studies of maize with fungal and viral pathogens (Cao *et al*., 2012; Chen *et al*., 2017a, 2017b; van der Linde *et al*., 2011, 2012; Zhu *et al*., 2014). Since the ten putative *ZmPAL* genes share high nucleotide sequence identity (Fig. S2), we cloned into the plasmid encoding the BMV vector a 153 bp fragment to target a sequence that is conserved between all of the *ZmPAL* genes (Fig. S2). To control for any effect of BMV infection on maize, we included a BMV vector containing a fragment (205 bp) of the gene sequence for the green fluorescent protein (BMV‐GFP) in all VIGS experiments. BMV virions were isolated from maize plants inoculated with BMV‐GFP or BMV‐PAL, and then mechanically inoculated to maize seedlings as previously described (Zhu *et al*., 2014).

**Figure 3:**
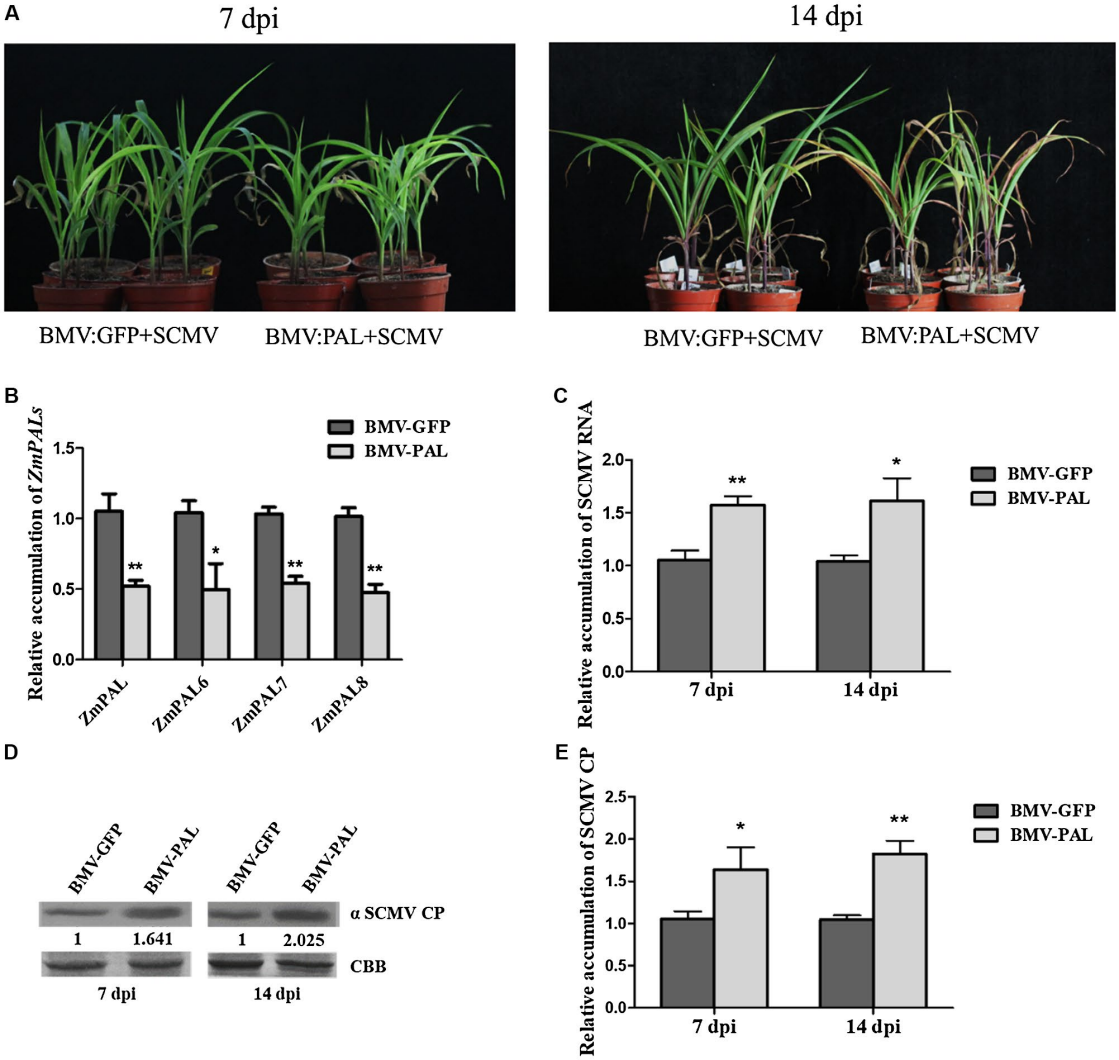
Knockdown of expression of *ZmPAL* genes in maize plants through BMV‐mediated VIGS increases SCMV multiplication and symptom severity. (A) SCMV induced more stunt and leaf early senescence symptoms on *ZmPAL‐*knockdown plants. Bars = 5 cm. (B) Silencing efficiency of *ZmPAL* genes in systemically infected leaves at 7 days post‐inoculation (dpi) measured by RT‐qPCR. (C) Relative accumulation of SCMV RNA in systemically infected leaves of the *ZmPAL*‐knockdown (BMV‐PAL) or control (BMV‐GFP) plants at 7 and 14 dpi. (D) Accumulation of SCMV CP in systemically infected leaves of the *ZmPAL*‐knockdown or control maize plants at 7 and 14 dpi measured by western blotting and visualized by ImageJ software. The numbers shown below the upper panel indicated the ratio of SCMV CP accumulation in the *ZmPAL*‐knockdown or control maize leaves. The amount of SCMV CP in the control (BMV‐GFP) maize plants is presented as 1. (E) Relative accumulation of SCMV CP in *ZmPAL*‐silenced leaves measured by ImageJ software. Three independent experiments were conducted with at least three biological replicates per treatment. Error bars represent the mean ± SE. Statistically significant differences are indicated (*P < 0.05, **P < 0.01) and were determined using Student’s *t*‐test.

At 10 dpi, plants infected with BMV‐PAL or BMV‐GFP were challenged with SCMV. At 7 days post‐SCMV infection, plants that had also been infected with BMV‐PAL were more stunted than plants infected with SCMV and the control VIGS vector BMV‐GFP (Fig. 3A). By 14 dpi, plants previously inoculated with BMV‐PAL showed necrosis in their upper leaves but necrosis did not occur in the plants inoculated with BMV‐GFP (Fig. 3A). RT‐qPCR showed that expression of *ZmPAL*, *ZmPAL6*, *ZmPAL7* and *ZmPAL8* was reduced by 40–60% in plants inoculated with BMV‐PAL (Fig. 3B), confirming that VIGS had been successful.

We analysed SCMV RNA and CP accumulation at 7 and 14 dpi. Results showed that the accumulation of SCMV genomic RNA in the *ZmPAL‐*silenced plants had surged by approximately 70% at 7 dpi and by 14 dpi was still 25% greater than levels in plants infected with the control vector, BMV‐GFP (Fig. 3C). Western blot results were consistent with the RT‐qPCR results and showed 1.6‐ and 2.0‐fold increases in SCMV CP accumulation at 7 and 14 dpi, respectively (Fig. 3D,E). These findings show that silencing *ZmPAL* genes expression enhances SCMV infection.

**Figure 4:**
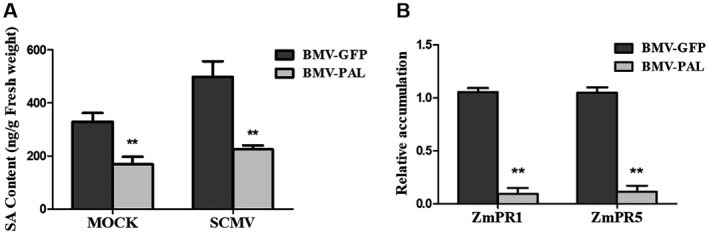
Knockdown of expression of *ZmPAL* genes in maize plants through BMV‐mediated VIGS compromises SA accumulation and SA‐regulated gene expression. Panel A shows SA accumulation data and *ZmPR1* and *ZmPR5* expression levels are shown in panel B. Three independent experiments were conducted with at least three biological replicates per treatment. Error bars represent the mean ± SE. Significant differences between BMV‐GFP and BMV‐PAL infected plants are indicated (**P < 0.01) and were determined using Student’s *t*‐test.

We measured SA accumulation and the expression of SA‐regulated genes in maize to establish whether SA accumulation in maize was dependent upon PAL activity and if SCMV infection triggered increased accumulation of SA. It was found that that the SA content and the accumulation of transcripts of the SA‐induced genes *ZmPR1* and *ZmPR5* were increased by SCMV infection. However, SA accumulation and induction of SA‐related genes were not as strongly increased in plants pre‐infected with the BMV‐PAL VIGS vector (Fig. 4). Thus, in maize SA biosynthesis can occur via a PAL‐dependent route and it is likely that SCMV‐induced SA accumulation is due to up‐regulation of the activity of the phenylpropanoid pathway.

### Exogenous application of SA decreased SCMV accumulation in *ZmPAL*‐silenced plants

**Figure 5:**
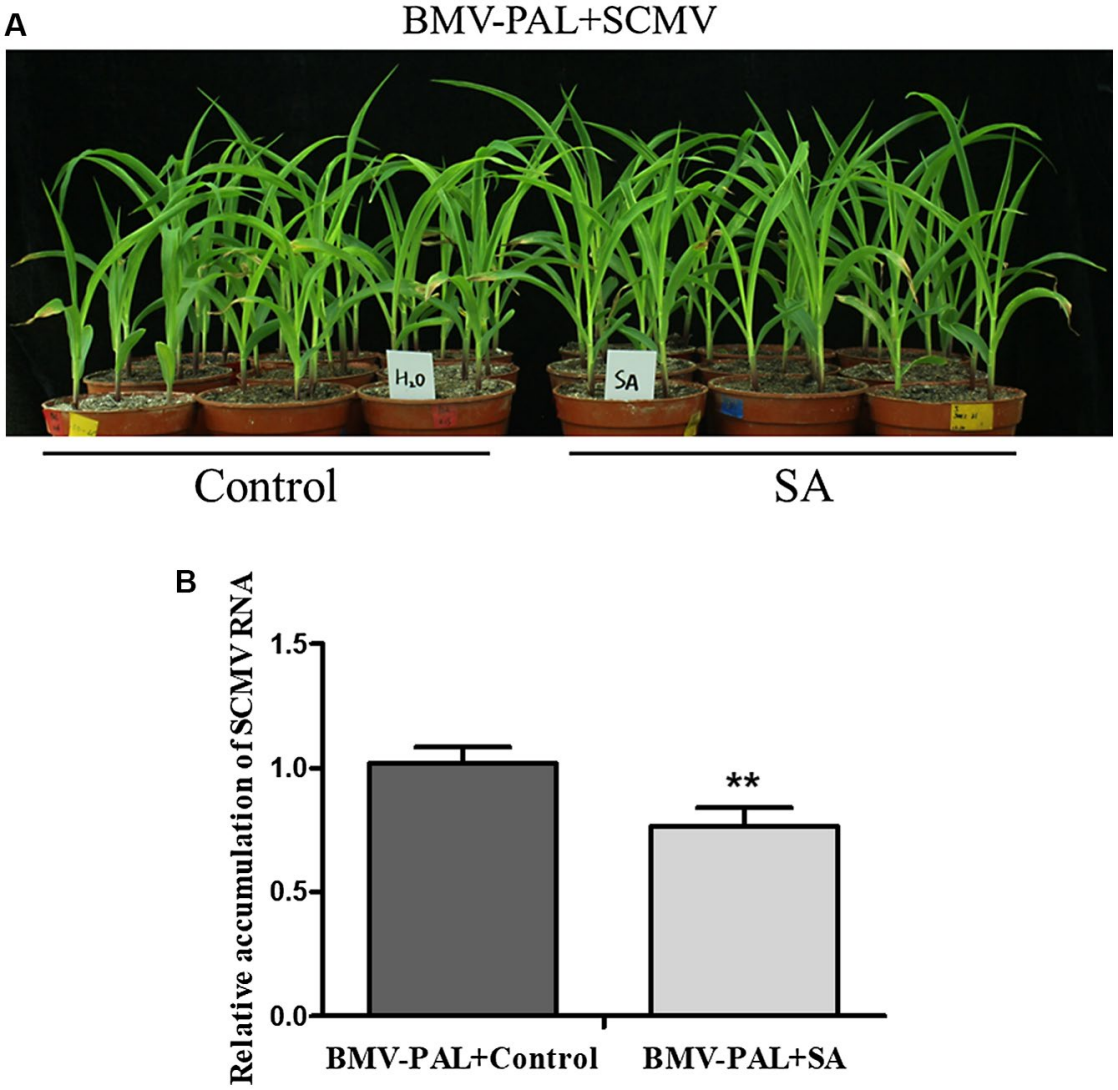
Exogenous application of SA restored the resistance to SCMV in *ZmPAL*‐knockdown plants. (A) Appearance of maize plants silenced for *ZmPAL* gene expression and treated with a control solution (water amended with Tween‐20: H_2_O) or 2 mM SA. Bars = 5 cm. The plants were at 7 days post‐inoculation of SCMV. (B) Lowered SCMV RNA accumulation in SA‐treated plants compared to sterile distilled water control‐treated plants. Three independent experiments were conducted with at least three biological replicates per treatment. Error bars represent the mean ± SE. Significant differences are indicated (**P < 0.01) and were determined using Student’s *t*‐test.

To confirm that decreased SA accumulation in *ZmPAL*‐silenced plants was responsible for the increase in SCMV RNA and CP accumulation (Fig. 3), SA levels in *ZmPAL*‐silenced plants were supplemented by administration of exogenous SA. *ZmPAL*‐silenced maize plants were used to determine the effects of *ZmPAL*s during SCMV infection (Fig. 5). *ZmPAL*‐silenced, SCMV‐infected plants sprayed with 2 mM SA were less stunted than the *ZmPAL*‐silenced, SCMV‐infected plants treated with a control solution (Fig. 5A). RT‐qPCR results showed that SCMV RNA accumulation in *ZmPAL*‐silenced SCMV‐infected plants was significantly decreased by application of exogenous SA (Fig. 5B).

### SCMV infection increases phenylpropanoid pathway activity and lignin accumulation

**Figure 6:**
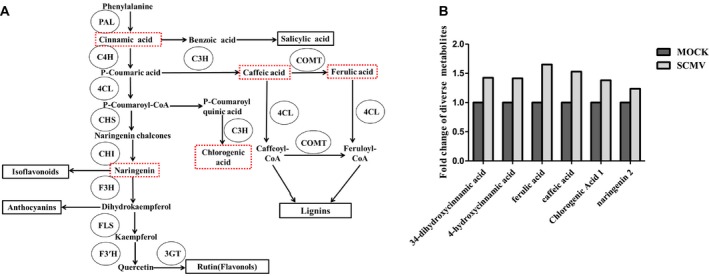
Metabolomic analysis showed increased accumulation of secondary metabolites in the phenylpropanoid pathway on SCMV infection. (A) The phenylpropanoid pathway. Up‐regulated metabolites are highlighted by red rectangles. PAL, phenylalanine ammonium lyase; C4H, cinnamic acid 4‐hydroxylase; 4CL, 4‐coumarate‐CoA ligase; CHS, chalcone synthase; CHI, chalcone isomerase; F3H, flavanone‐3‐hydroxylase; FLS, flavonol synthase; F3′H, flavonoid‐3′‐hydroxylase; 3GT, flavonoid 3‐*O*‐glucosyltransferase; C3H, *p*‐coumarate‐3‐hydroxylase; COMT, caffeate *O*‐methyltransferase. (B) Relative accumulation levels of secondary metabolites generated via the phenylpropanoid pathway in SCMV‐infected or mock‐inoculated maize plants.

PAL catalyses the first step of the phenylpropanoid pathway, which is required for biosynthesis of many phenolic secondary metabolites (Fig. 6A: modified from Zhao *et al*., 2013). To determine how virus infection alters the production of metabolites generated through the phenylpropanoid pathway, we conducted a metabolomic analysis of SCMV‐infected and mock‐inoculated maize leaves. The results showed that biosynthesis of 3,4‐dihydroxycinnamic acid, 4‐hydroxycinnamic acid, ferulic acid, caffeic acid, chlorogenic acid and naringenin was significantly up‐regulated in SCMV‐infected leaves (Fig. 6B). This suggests that the increased expression of *ZmPAL* genes on SCMV infection causes a general increase in the biosynthetic activity of the phenylpropanoid pathway.

**Figure 7:**
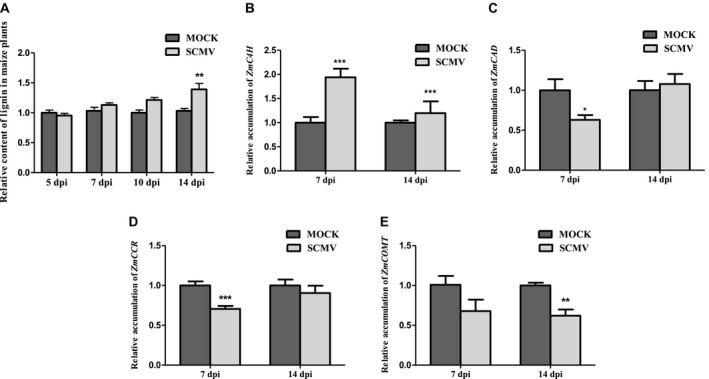
SCMV infection induces lignin accumulation and altered expression of genes related to lignin synthesis in maize. (A) Relative accumulation of lignin in SCMV‐infected or mock‐inoculated maize plants. (B)–(E) Relative expression levels of transcripts for genes related to lignin biosynthesis (*ZmC4H*, *ZmCAD*, *ZmCCR* and *ZmCOMT*) in SCMV‐infected or mock‐inoculated maize plants. Three independent experiments were conducted with at least three biological replicates per treatment. Error bars represent the mean ± SE. Significant differences are indicated (*P < 0.05, **P < 0.01, ***P < 0.001) and were determined using Student’s *t*‐test.

Lignin is produced through the activity of the phenylpropanoid pathway. This biopolymer plays an important role in strengthening cell walls and its synthesis is stimulated by stress (An and Mou, 2011; Gayoso *et al*., 2010; Xu *et al*., 2011; Yu and Jez, 2008). Since SCMV infection causes increased activity of the phenylpropanoid pathway, we investigated whether SCMV infection increased lignin biosynthesis and the expression of genes related to lignin biosynthesis. We found that the lignin content of SCMV‐infected plants was similar to that in mock‐inoculated plants for up to 10 dpi but became significantly greater by 14 dpi (Fig. 7A).

Expression of four genes encoding key enzymes in lignin biosynthesis (Barrière *et al*., 2004; Guillaumie *et al*., 2007) was analysed by RT‐qPCR. The expression of *Cinnamate 4‐hydroxylase* (*ZmC4H*, Zm00001d009858) was significantly increased in SCMV‐infected plants at 7 and 14 dpi (Fig. 7B). Transcript levels for *Cinnamyl alcohol dehydrogenase* (*ZmCAD*, Zm00001d015618) and *Cinnamyl alcohol dehydrogenase* (*ZmCCR*, Zm00001d032152) were down‐regulated significantly in SCMV‐infected maize plants at 7 dpi followed by a recovery by 14 dpi (Fig. 7C,D). Expression of *Caffeic acid 3‐O‐methyltransferase* (*ZmCOMT*, Zm00001d049541) was significantly down‐regulated at 14 dpi (Fig. 7E). Overall, these results show that SCMV infection alters the expression of genes related to lignin biosynthesis in a complex fashion.

### Decreasing expression of *ZmPALs* using VIGS reduced lignin content and altered the expression of genes related to lignin biosynthesis

**Figure 8:**
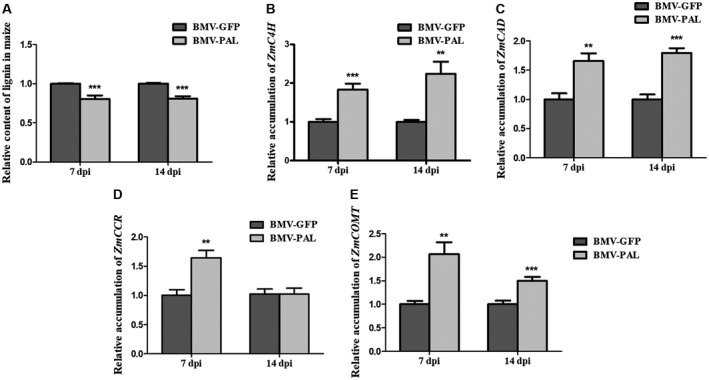
Knockdown of expression of *ZmPAL* gene in maize plants decreased lignin content and altered lignin‐related gene expression. (A) Relative accumulation of lignin in SCMV infected *ZmPAL*‐knockdown (BMV‐PAL) or vector control (BMV‐GFP) maize plants. (B)–(E) RT‐qPCR data for relative expression levels of transcripts encoding enzymes of lignin biosynthesis‐related genes (*ZmC4H*, *ZmCAD*, *ZmCCR* and *ZmCOMT*) in maize plants infected with SCMV as well as BMV‐derived vectors BMV‐PAL or BMV‐GFP (control VIGS vector). Three independent experiments were conducted with at least three biological replicates per treatment. Error bars represent the mean ± SE. Significant differences are indicated (**P < 0.01, ***P < 0.001) and were determined using Student’s *t*‐test.

In this study we analysed lignin content and the expression of lignin biosynthesis‐related genes in *ZmPAL*‐silenced and non‐silenced maize plants on SCMV infection. *ZmPAL*‐silenced, SCMV‐infected maize plants accumulated less lignin than non‐silenced, SCMV‐infected plants (Fig. 8A).

Transcripts for *ZmC4H*, *ZmCAD*, *ZmCCR* and *ZmCOMT* were all up‐regulated by about 2‐fold in the *ZmPAL*‐silenced SCMV‐infected plants compared with that in the non‐silenced SCMV‐infected plants at 7 dpi (Fig. 8B–E). At 14 dpi, the expression levels of *ZmC4H*, *ZmCAD* and *ZmCOMT* in the *ZmPAL*‐silenced SCMV‐infected plants were still 1.5‐ to 2‐fold higher than in the non‐silenced SCMV‐infected control plants (Fig. 8B–E). The expression level of *ZmCCR* in the *ZmPAL*‐silenced SCMV‐infected plants returned to a level similar to that in the control plants (Fig. 8D).

## Discussion

Understanding the pathways controlling pathogen resistance is crucial for developing new disease management strategies. Through analyses of metabolites and gene expression during SCMV infection, we have determined that in maize SA biosynthesis is dependent upon PAL activity and the activation of the phenylpropanoid pathway. We have shown that inhibition of SCMV infection can be artificially generated by treating plants with exogenous SA prior to challenge with the virus. Our results also show that the increase in SA accumulation triggered by SCMV infection serves to confer resistance to SCMV via limiting virus titre and symptom severity.

### SA inhibits the accumulation of SCMV in maize

SA inhibits the replication and movement of several viruses in tobacco, *Arabidopsis* and cucurbits, all of which are dicots (Murphy and Carr, 2002; Naylor *et al*., 1998; Wong *et al*., 2002). However, the antiviral defence role of SA and the effects of virus infection on SA biosynthesis are less well explored in monocots such as rice and maize (Klessig *et al*., 2018; Takatsuji, 2014; Vlot *et al*., 2009). We found that pre‐treatment of maize plants with SA inhibits the accumulation of viral RNA and CP in systemically infected tissue. Consistent with earlier reports on SA treatment of virus‐susceptible *A. thaliana*, tobacco, *N. benthamiana* and cucumber plants, the effect of the exogenous treatment is to delay the onset of disease and decrease virus titre, rather than engender complete resistance to the virus (Lee *et al*., 2011; Mayers *et al*., 2005; Murphy and Carr, 2002; Naylor *et al*., 1998; Smith‐Becker *et al*., 2003; Wong *et al*., 2002).

SCMV infection induced endogenous SA accumulation in maize plants and increased expression of the SA‐regulated genes *ZmPR1* and *ZmPR5*. Increases in SA accumulation have been observed in several compatible plant–virus interactions (Krečič‐Stres *et al*., 2005; Love *et al*., 2005; Whitham *et al*., 2003; Wu *et al*., 2013b; Zhou *et al*., 2014). Results of a previous study suggested an increase in SA in maize infected by SCMV (Wu *et al*., 2013b) but the role of SA in SCMV infection remains unclear. Interestingly, SCMV‐induced SA accumulation was greater at 7 dpi than at 14 dpi, suggesting that accumulation of this metabolite declines once the most active period of viral spread has concluded. It is also conceivable that maize plants are in some way adjusting their allocation of resources to balance growth with maintenance of immunity to the virus. Since supplementing *ZmPALs*‐silenced SCMV‐infected plants with exogenous SA decreased the accumulation of SCMV, it appears that the induction of endogenous SA biosynthesis helps to limit the virulence of SCMV. This may represent a limited form of host resistance (or tolerance) to SCMV. Alternatively, it may be that the virus has evolved the ability to stimulate SA biosynthesis in order to moderate its own accumulation and prevent unnecessary damage to the host.

In previous reports on CMV, CaMV and the potyviruses tobacco etch virus and turnip mosaic virus the respective viral RNA silencing suppressors (2b, P6 and P1/HC‐Pro) were shown to be involved in manipulation of SA‐induced defence (Alamillo *et al*., 2006; Ji and Ding, 2001; Love *et al*., 2012; Poque *et al*., 2018; Zhou *et al*., 2014). Future studies are needed to determine if the HC‐Pro or P1/HC‐Pro proteins of SCMV enable the virus to subvert SA‐induced resistance in maize and regulate the initial increase and subsequent decline in virus‐induced SA biosynthesis.

### *ZmPALs* are required for SA biosynthesis and may positively regulate SA‐dependent defence signalling in maize

Like PALs in *Arabidopsis* and tomato (Guo and Wang, 2009), *Bambusa oldhamii* (Hsieh *et al*., 2010), *Phyllostachys edulis* (Gao *et al*., 2012), pepper (Kim and Hwang, 2014) and rice (Duan *et al*., 2014), ZmPALs are encoded by a multi‐gene family. Increased expression of *PAL* genes is induced by fungal and bacterial infections in dicotyledonous plants (Kim and Hwang, 2014; MacDonald and D′Cunha, 2007; Shine *et al*., 2016). Fewer studies have investigated *PAL* gene induction by viruses but rice *PAL* genes were reported to be up‐regulated by rice yellow mottle virus infection (Delalande *et al*., 2005). Our RT‐qPCR and RNA‐Seq results show that expression of a subset of *ZmPAL* genes family members was significantly up‐regulated during SCMV infection. VIGS‐mediated inhibition of *ZmPAL* genes expression shows that in maize the increased expression of these *ZmPAL* genes and the consequent increase in activity of the phenylpropanoid pathway is required for SA biosynthesis and activation of SA‐dependent defensive signalling. Thus, maize differs from *Arabidopsis* or barley, in which the isochorismate pathway is the predominant biosynthetic route for SA production (Hao *et al*., 2018; Wildermuth *et al*., 2001), or soybean, in which the phenylpropanoid and isochorismate pathways are equally important (Shine *et al*., 2016). It appears that the phenylpropanoid pathway is the major route for biosynthesis of SA in maize.

### SCMV induced lignin accumulation

SCMV infection triggered increased production of multiple phenylpropanoid pathway metabolites including 3,4‐dihydroxycinnamic acid, 4‐hydroxycinnamic acid, ferulic acid, caffeic acid, chlorogenic acid and naringenin. Significantly, ferulic acid, caffeic acid and chlorogenic acid are the intermediates of lignin biosynthesis (Barrière *et al*., 2004; Shine *et al*., 2016; Silverman *et al*., 1995; Zhao *et al*., 2013). Consistent with this, our results showed that SCMV‐infected maize plants accumulated more lignin but this occurred at a later stage of infection than peak accumulation of SA. As expected, using VIGS to decrease *ZmPAL* transcripts accumulation compromised lignin biosynthesis and altered the expression of lignin biosynthesis‐related genes.

Lignin is a major strengthening component of plant secondary cell walls and inhibits the spread of some pathogens (Huang *et al*., 2010). Conceivably, the elevated lignin content seen in SCMV‐infected maize might help to strengthen or modify maize cell walls and thereby impede SCMV cell‐to‐cell movement via plasmodesmata. However, since the increase in lignin is only evident by 14 dpi, when the virus will have already spread systemically, it is unlikely that lignin plays a critical role in restricting the dissemination of SCMV through the host.

In conclusion, our study indicates that *ZmPAL* genes are required for SA biosynthesis and activation of resistance responses to virus infections in maize. Knocking down expression of *ZmPAL* genes in maize resulted in increased SCMV accumulation and pathogenicity, decreased SA biosynthesis, diminished expression of the SA‐responsive marker genes, *ZmPR1* and *ZmPR5*, and inhibited lignin accumulation. These effects were reversed by addition of exogenous SA. Thus, PAL and the phenylpropanoid pathway provide the bulk of SA in maize and are required for the functioning of SA‐dependent defensive signalling in this important crop plant species. Importantly, our experiments with SA‐treated, *ZmPAL*‐silenced plants appear to explain a previously puzzling observation. Specifically, that in certain compatible virus–plant interactions, SA biosynthesis and/or SA‐regulated gene expression is up‐regulated (e.g. see Whitham *et al*., 2003 or Zhou *et al*., 2014). Our results indicate that this is a mechanism that limits the damage caused by a virus but whether this is primarily a limited defence response by the host or a viral mechanism to modulate replication remains to be seen.

## Experimental Procedures

### Plant cultivation and virus inoculation

Maize (*Z. mays* L. inbred lines B73 and Va35) and *N. benthamiana* Domin. plants were grown in growth chambers set at 24/22 °C (day/night) with a 16/8 h (light/dark) photoperiod as described previously (Chen *et al*., 2017a). SCMV‐BJ was propagated in maize plants and homogenates from SCMV‐infected maize leaves were used to inoculate the first true leaf of 8‐day‐old maize seedlings as described (Cao *et al*., 2012; Zhu *et al*., 2014). Mechanical inoculation of maize seedlings was done by rubbing the first true leaf with the virus‐containing homogenate or 0.1 M phosphate buffer (pH 7.0) as a mock‐inoculation control.

### SA measurements

SA was extracted from upper leaves of mock‐inoculated or SCMV‐infected plants and measured by ultra‐high performance liquid chromatography–triple quadrupole mass spectrometry (UPLC‐MS/MS) following the method of Pan *et al. *(2010) at the mass spectrometry centre of the College of Biological Sciences, China Agricultural University. There were three biological repeats for each treatment investigated.

### Total RNA extraction and RT‐qPCR

Total RNA was isolated using TRIzol reagent (Tiangen, Beijing, China) followed by RNase‐free DNase I treatment (TaKaRa, Dalian, China). First‐strand cDNA was synthesized using 2.0 μg total RNA per 20 μL reaction and an oligo (dT) primer. Ten‐fold diluted cDNA, a set of gene‐specific primers (Table S1) and a FastSYBR mixture (CWBIO, Beijing, China) were mixed for qPCR to determine the accumulation level of SCMV RNA and maize genes on an ABI 7500 Real Time PCR system (Applied Biosystems Inc., Foster City, CA, USA). The expression level of *ZmUbi* mRNA was determined and used as an internal control. The relative expression level of each gene was calculated using the 2^–ΔΔCt^ method (Livak and Schmittgen, 2001). Differences between the treatments were then analysed using Student’s *t*‐test. All experiments were carried out at least three times.

### Western blot assay

Total leaf protein preparation and electrophoresis were performed as previously described (Cao *et al*., 2012). Detection of SCMV in the samples was performed using an antiserum specific for SCMV CP with a secondary antibody conjugated to alkaline phosphatase with phosphatase activity detected using nitroblue tetrazolium/5‐bromo‐4‐chloro‐3‐indolylphosphate (NBT/BCIP) (Roche) (Xia *et al*., 2016). Quantification of CP was estimated using ImageJ software (http://imagej.net/) as described by Wyrsch *et al. *(2015).

### SA treatment

For treatment of maize plants with exogenous SA, two‐leaf‐stage maize plants were sprayed with 2 mM SA dissolved in sterile distilled water containing 0.2% (v/v) Tween‐20 as a wetting agent. The control treatment was spraying with sterile distilled water containing 0.2% (v/v) Tween‐20. The experiment was carried out at least three times and 18 plants were used for each treatment. On *ZmPAL*‐silenced plants, SA treatment was followed 2 days later by SCMV inoculation. Symptoms were observed and SCMV RNA and CP accumulation were measured 7 days after inoculation.

### Sequence accessions and phylogenetic analysis

Putative *ZmPAL* genes were obtained from the updated *Z. mays* B73 genome website (AGPv4,  http://ensembl.gramene.org/Zea_mays/Info/Index). *PAL* genes of other plant species were retrieved from the GenBank and used as references. Multiple sequence alignments were performed by DNAMAN 7.0 (Lynnon Biosoft, San Ramon, CA, USA). Phylogenetic trees were constructed using the neighbour‐joining method (1000 replicates) in the MEGA5 program using the aligned nucleotide or amino acid sequences generated using Clustal X software (Tamura *et al*., 2011).

### Virus‐induced silencing of *ZmPAL* gene expression

The BMV‐derived VIGS system was described previously (Zhu *et al*., 2014). A DNA fragment of 153 bp representing a sequence conserved between *ZmPAL* genes was amplified using primers ZmPAL‐silencing‐F and ZmPAL‐silencing‐R (Supplementary Fig. S2; Supplementary Table S1). Amplicons were digested with *Nco*I and *Bln*I (NEB Inc., Beverly, MA, USA), and inserted into the pC13/F3‐13m vector (Zhu *et al*., 2014). The resulting vector (pC13/F3‐13m:ZmPAL) was sequenced before further use.

The construct pC13/F3‐13m:ZmPAL was introduced into *Agrobacterium tumefaciens* strain C58C1. *Agrobacterium tumefaciens* cultures carrying pC13/F1 + 2, pC13/F3‐13m:ZmPAL or pC13/F3‐13m:GFP were grown, pelleted and resuspended individually in infiltration buffer (10 mM MgCl_2_, 10 mM MES pH 5.7 and 200 μM acetosyringone in sterile water) till OD_600_ = 2.0. The *A. tumefaciens* cultures harbouring pC13/F1 + 2 were mixed with either pC13/F3‐13m:ZmPAL or pC13/F3‐13m:GFP in 1:1 (v/v) ratios before infiltration into *N. benthamiana* leaves. At 3 dpi, the agro‐infiltrated *N. benthamiana* leaves were harvested and used for BMV virion purification. Approximately 20 μg partially purified BMV virion was rub‐inoculated to a 1‐week‐old Va35 maize seedling. More than 20 plants were used for each treatment and the inoculated plants were grown inside a growth chamber at 18/20 °C (day/night) for 7 days before being challenged again with SCMV as described (Zhu *et al*., 2014). Systemically infected leaves (or equivalent leaves from mock‐inoculated plants) from BMV‐PAL (VIGS vector) or BMV‐GFP (control vector) inoculated plants were harvested from individually plants at 7 and 14 dpi, and subjected to RT‐qPCR.

### Lignin analysis using the acetyl bromide method

Lignin quantification was performed as previously described (Chezem *et al*., 2017) with specific modifications. The first SCMV‐infected systemically infected leaves (i.e. leaves adjacent to SCMV‐inoculated leaves; 1 SL) or equivalent leaves from mock‐inoculated plants were harvested from individual maize plants and immediately ground in liquid nitrogen inside a ball mill with a 5 mm stainless steel bead for 2 min. The ground samples were ultrasonicated for 15 min in a mixture of twice with 1 mL methanol, twice with phosphate‐buffered saline pH (7.0) containing 0.1% (v/v) Tween 20, twice with 1 mL 95% ethanol, twice with 1 mL (1:1) chloroform:methanol and twice with 1 mL acetone. The samples were centrifuged at 16 000 *g* for 10 min and the pellets dried at 50 °C. The dried pellets were individually ground for 10 min with 1 mm zirconia beads (10–15 beads) inside ball mills set at 25 Hz, dissolved in 1.5 mL of 25% (v/v) acetyl bromide (Sigma‐Aldrich, St. Louis, MO, USA) in glacial acetic acid, and heated at 50 °C for 2 h with occasional shaking. The ball mills were cooled on ice and 125 μL of each sample was transferred into a centrifuge tube containing 250 μL of a mixture of 5 M hydroxylamine‐HCl (Sigma‐Aldrich, St. Louis, MO, USA) and 2 M NaOH (1:9, v/v), and 500 μL of glacial acetic acid. The absorbance at 280 nm was determined for each sample using a microplate reader (SpectraMax i3x, Molecular Devices, Arlington, Texas, USA).

## Conflict of Interest

The authors declare that they have no conflict of interest.

## Supporting information


**Fig. S1** SA treatment did not alter plant growth or development in maize.

**Fig. S2** Multiple nucleotide sequences alignment showed high identity of *ZmPAL*s genes.

**Fig. S3** Multiple amino acid sequences alignment showed high identity (79.72%) of *ZmPAL*s genes.

**Fig. S4** Phylogenetic tree of *ZmPAL*s genes based on amino acid sequences.

**Fig. S5** Multiple amino acid sequences alignment showed high identity (11.5–99.4%) of PAL proteins encoded by *PAL* gene families from *Zea mays*, *Arabidopsis thaliana*, *Brachypodium distachyon*, *Hordeum vulgare*, *Oryza sativa* and *Glycine max*.

**Fig. S6** Phylogenetic tree showing that monocot  and dicot encoded *PAL *genes cluster separately.

**Fig. S7** RNA Seq analysis identified *ZmPAL*s transcripts that are induced by SCMV infection.

**Table S1** Primers used in this study.
