# Supplementary material for: Maize phenylalanine ammonia‐lyases contribute to resistance to Sugarcane mosaic virus infection, most likely through positive regulation of salicylic acid accumulation
Source: Mol Plant Pathol. 2019 Sep 5;20(10):1365–78. doi: 10.1111/mpp.12817 (PMC6792131; doi:10.1111/mpp.12817)
Supplement: Supplementary file 1 — Fig. S1 SA treatment did not alter plant growth or development in maize. [file MPP-20-1365-s001.pdf]

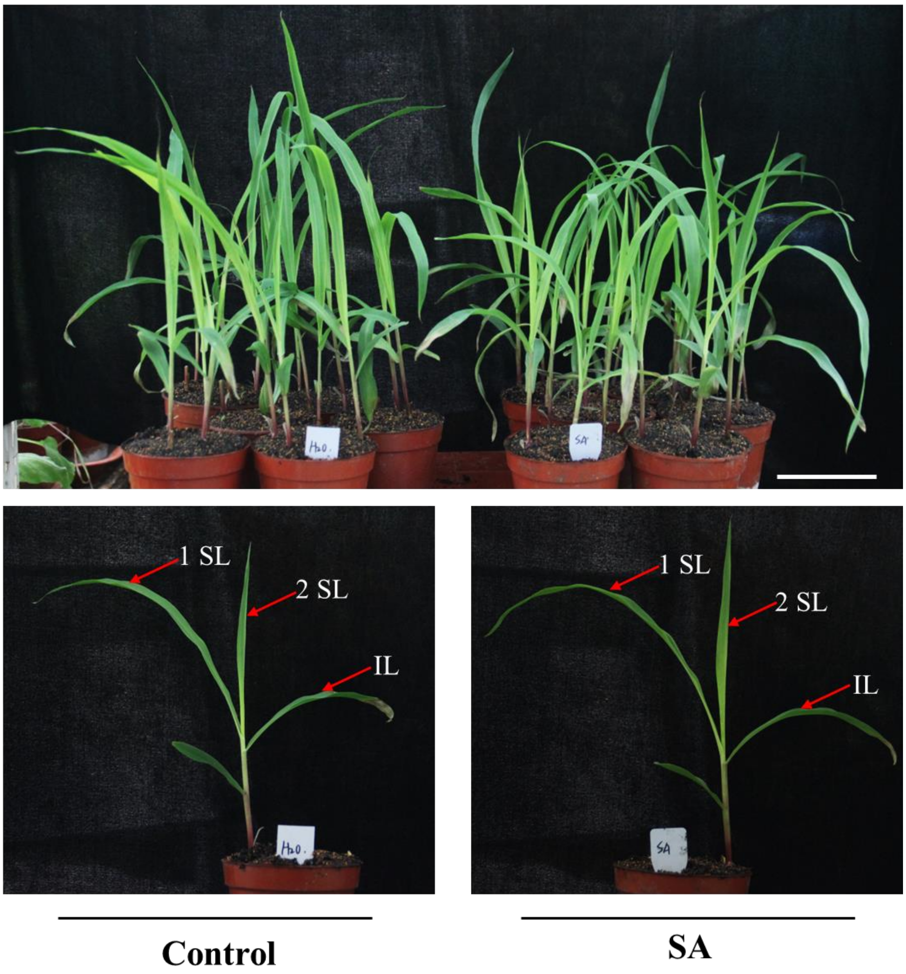

Fig. S1. SA treatment did not alter plant growth or development in maize. At 9 days following SA treatment there were no obvious effects on height or leaf developmental on maize plants. IL, Inoculated leaf; 1 SL, adjacent (first) non-inoculated, systemically-infected leaf; 2 SL, second non-inoculated, systemically-infected leaf. Scale bar = 10 cm.
