# Supplementary material for: Maize phenylalanine ammonia‐lyases contribute to resistance to Sugarcane mosaic virus infection, most likely through positive regulation of salicylic acid accumulation
Source: Mol Plant Pathol. 2019 Sep 5;20(10):1365–78. doi: 10.1111/mpp.12817 (PMC6792131; doi:10.1111/mpp.12817)
Supplement: Supplementary file 2 — Fig. S2 Multiple nucleotide sequences alignment showed high identity of ZmPALs genes. [file MPP-20-1365-s002.pdf]

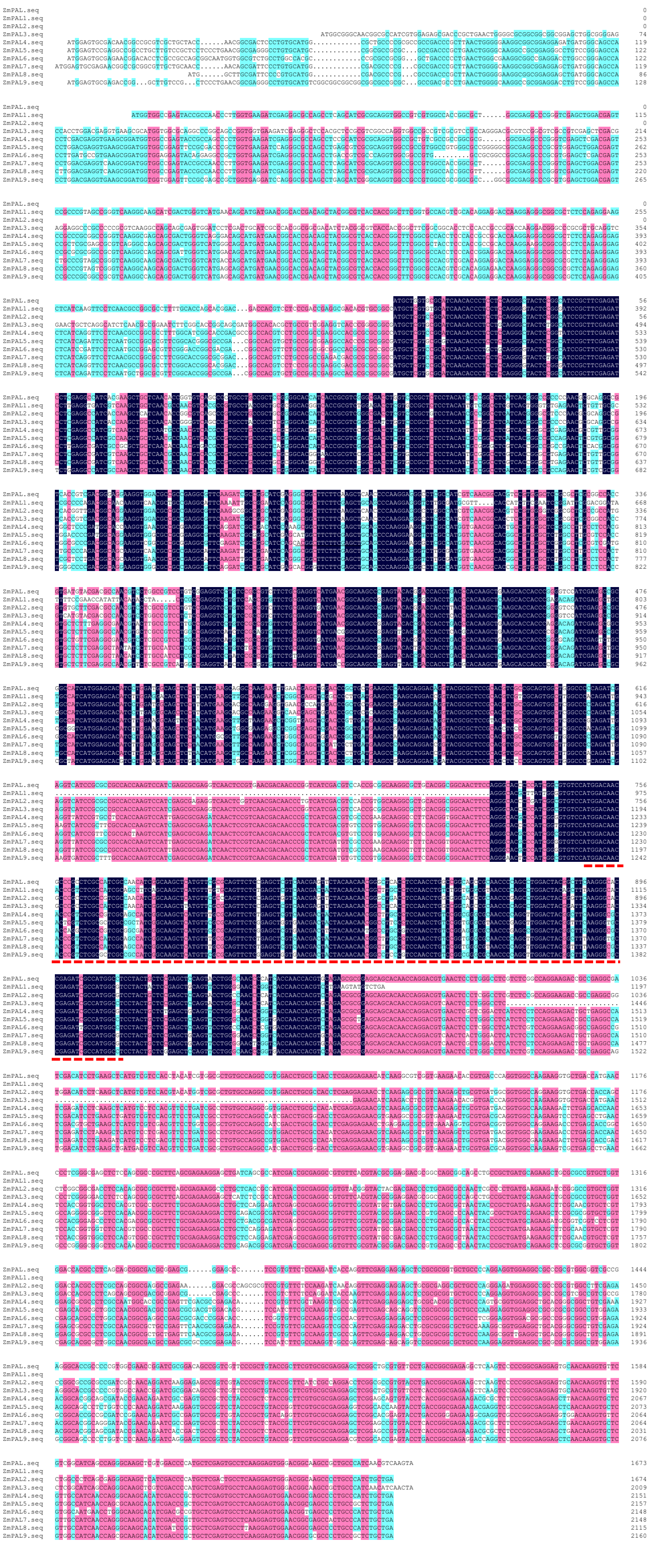

Fig. S2 Multiple nucleotide sequences alignment showed high identity of *ZmPAL* genes. The red

dotted line indicates the 153 bp conserved *ZmPAL* sequence that was used to knock down *ZmPAL* gene expressions using VIGS.
