# Supplementary material for: Maize phenylalanine ammonia‐lyases contribute to resistance to Sugarcane mosaic virus infection, most likely through positive regulation of salicylic acid accumulation
Source: Mol Plant Pathol. 2019 Sep 5;20(10):1365–78. doi: 10.1111/mpp.12817 (PMC6792131; doi:10.1111/mpp.12817)
Supplement: Supplementary file 3 — Fig. S3 Multiple amino acid sequences alignment showed high identity (79.72%) of ZmPALs genes. [file MPP-20-1365-s003.pdf]

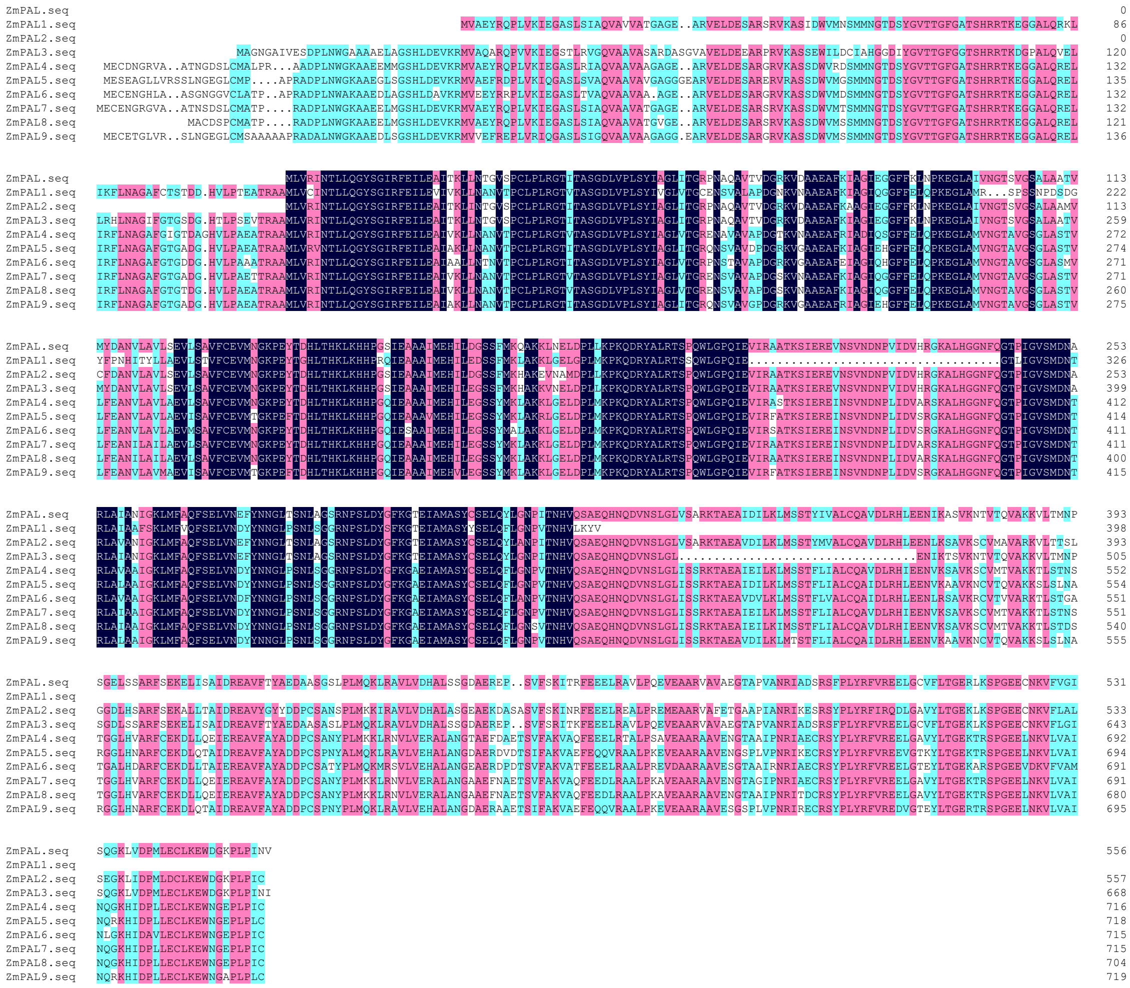

Fig. S3 Multiple amino acid sequences alignment showed high identity (79.72%) of *ZmPAL* genes.
