# Supplementary material for: Maize phenylalanine ammonia‐lyases contribute to resistance to Sugarcane mosaic virus infection, most likely through positive regulation of salicylic acid accumulation
Source: Mol Plant Pathol. 2019 Sep 5;20(10):1365–78. doi: 10.1111/mpp.12817 (PMC6792131; doi:10.1111/mpp.12817)
Supplement: Supplementary file 4 — Fig. S4 Phylogenetic tree of ZmPALs genes based on amino acid sequences. [file MPP-20-1365-s004.pdf]

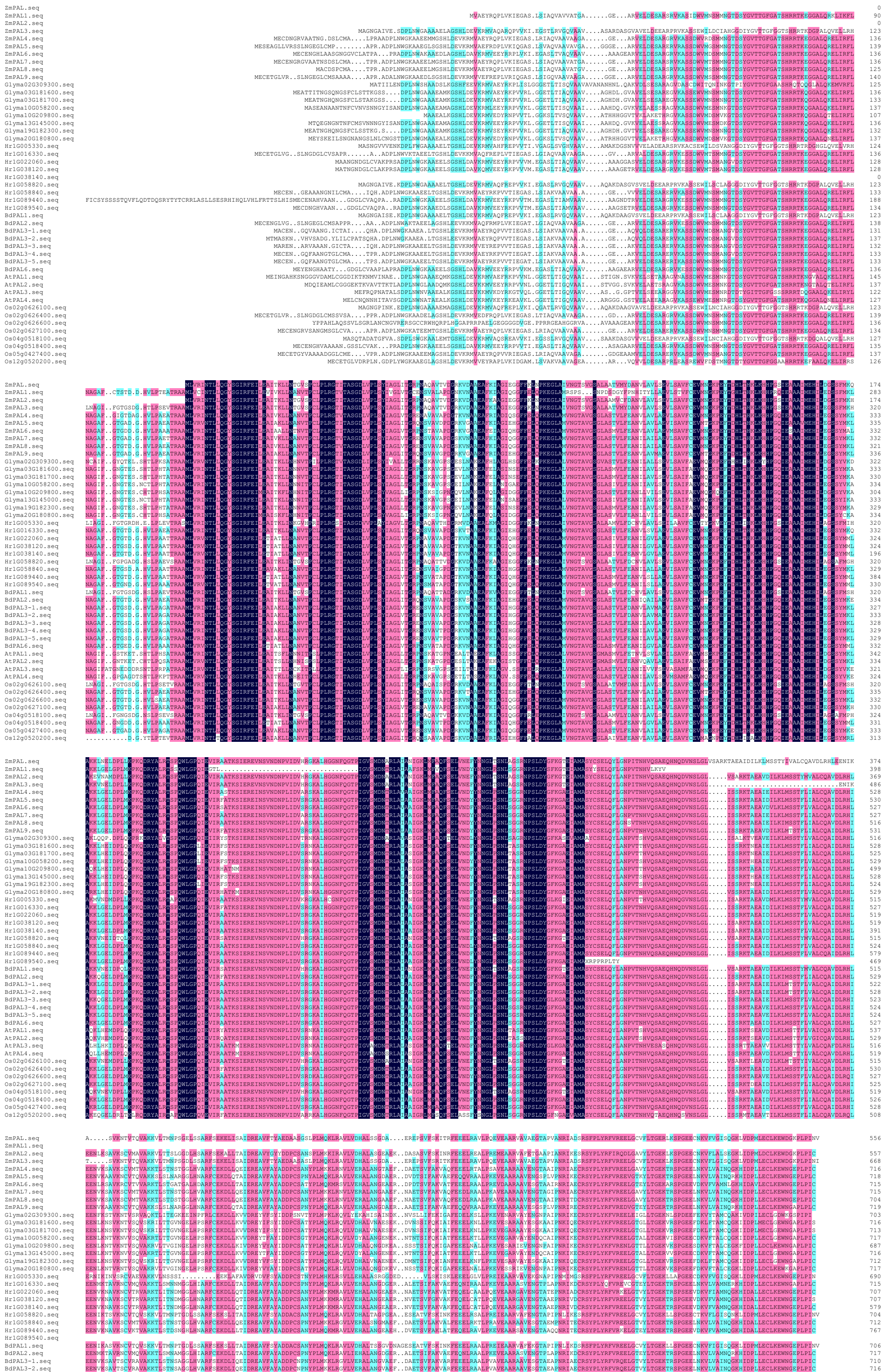

Fig. S4. Multiple amino acid sequences alignment showed high identity (11.5 % to 99.4%) of PAL proteins encoded by *PAL* gene families from *Zea mays*, *Arabidopsis thaliana*, *Brachypodium distachyon*, *Hordeum vulgare*, *Oryza sativa* and *Glycine max.*
