# Supplementary material for: Maize phenylalanine ammonia‐lyases contribute to resistance to Sugarcane mosaic virus infection, most likely through positive regulation of salicylic acid accumulation
Source: Mol Plant Pathol. 2019 Sep 5;20(10):1365–78. doi: 10.1111/mpp.12817 (PMC6792131; doi:10.1111/mpp.12817)
Supplement: Supplementary file 5 — Fig. S5 Multiple amino acid sequences alignment showed high identity (11.5–99.4%) of PAL proteins encoded by PAL gene families from Zea mays, Arabidopsis thaliana, Brachypodium distachyon, Hordeum vulgare, Oryza sativa and Glycine max. [file MPP-20-1365-s005.pdf]

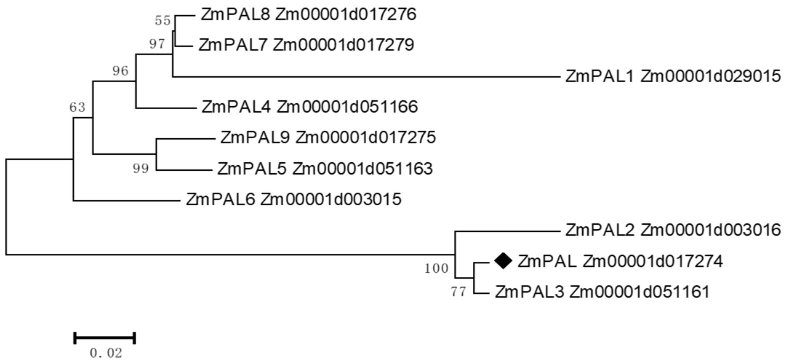

Fig. S5 Phylogenetic tree of *ZmPAL* genes based on amino acid sequences encoded by each gene family member. Bootstrap values lower than 50% were not shown, and the black square indicated the ZmPAL.
