# Supplementary material for: Maize phenylalanine ammonia‐lyases contribute to resistance to Sugarcane mosaic virus infection, most likely through positive regulation of salicylic acid accumulation
Source: Mol Plant Pathol. 2019 Sep 5;20(10):1365–78. doi: 10.1111/mpp.12817 (PMC6792131; doi:10.1111/mpp.12817)
Supplement: Supplementary file 6 — Fig. S6 Phylogenetic tree showing that monocot and dicot encoded PAL genes cluster separately. [file MPP-20-1365-s006.pdf]

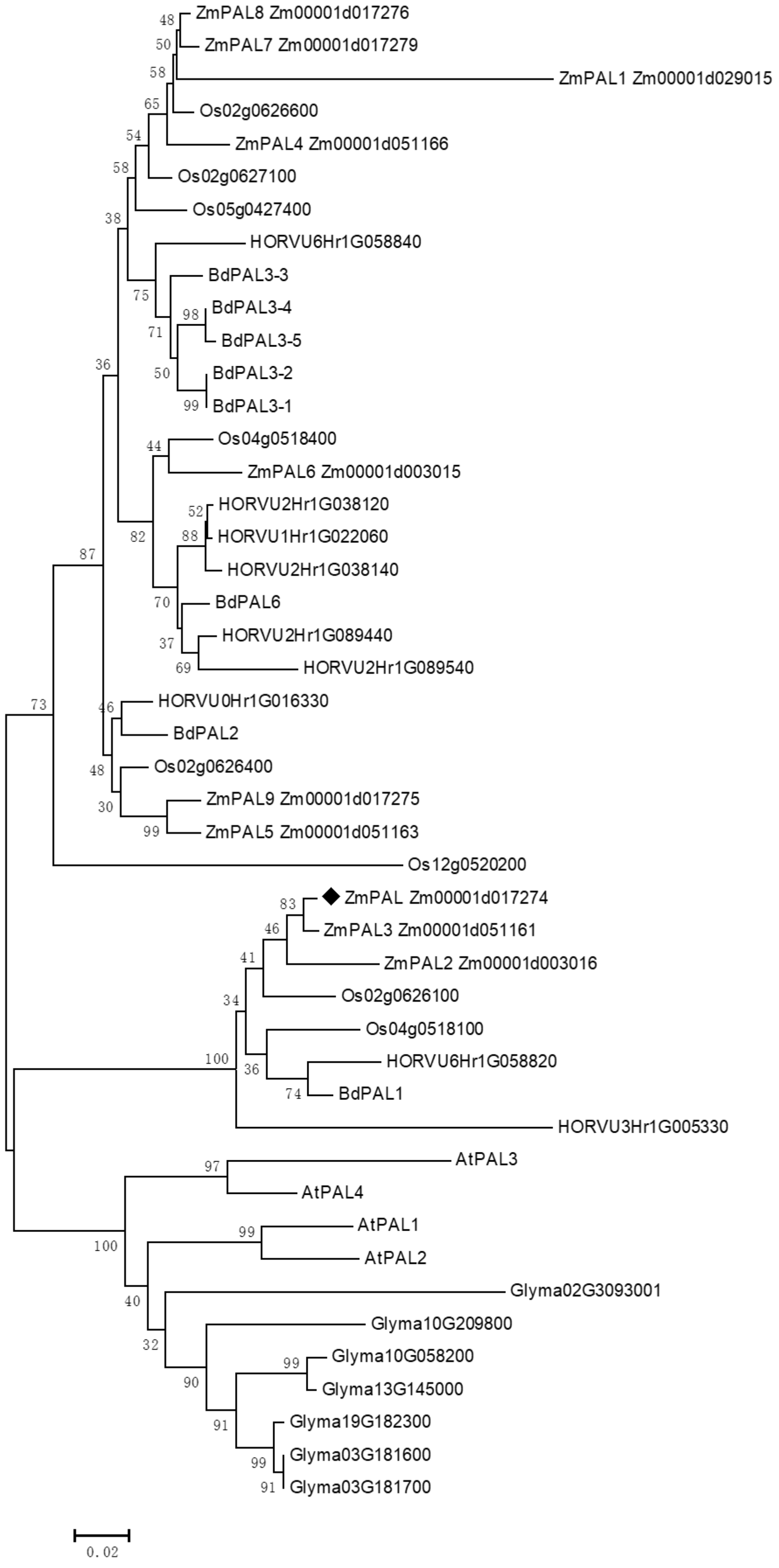
ZmPAL8 Zm00001d017276
ZmPAL7 Zm00001d017279
ZmPAL1 Zm00001d029015
Os02g0626600
ZmPAL4 Zm00001d051166
Os02g0627100
Os05g0427400
HORVU6Hr1G058840
BdPAL3-3
BdPAL3-4
BdPAL3-5
BdPAL3-2
BdPAL3-1
Os04g0518400
ZmPAL6 Zm00001d003015
HORVU2Hr1G038120
HORVU1Hr1G022060
HORVU2Hr1G038140
BdPAL6
HORVU2Hr1G089440
HORVU2Hr1G089540
HORVU0Hr1G016330
BdPAL2
Os02g0626400
ZmPAL9 Zm00001d017275
ZmPAL5 Zm00001d051163
Os12g0520200
ZmPAL Zm00001d017274
ZmPAL3 Zm00001d051161
ZmPAL2 Zm00001d003016
Os02g0626100
Os04g0518100
HORVU6Hr1G058820
BdPAL1
HORVU3Hr1G005330
AtPAL3
AtPAL4
AtPAL1
AtPAL2
Glyma02G3093001
Glyma10G209800
Glyma10G058200
Glyma13G145000
Glyma19G182300
Glyma03G181600
Glyma03G181700
0.02

Fig. S6. Phylogenetic tree showing that monocot- and dicot-encoded *PAL* genes cluster separately. Phylogenetic tree based on the encoded amino acid sequences was constructed using the Neighbor-Joining method (1000 replicates) implemented in the MEGA5 program with sequence alignments generated by Clustal X. Abbreviations: Zm, *Zea mays*; At, *Arabidopsis thaliana*; Bd, *Brachypodium distachyon*; Hv, *Hordeum vulgare*; Os, *Oryza sativa*; Gm, *Glycine max*. Database accessions for sequences used in this study are Zm00001d029015 (chromosome 1), Zm00001d003015 (chromosome 2), Zm00001d003016 (chromosome 2), Zm00001d051166 (chromosome 4), Zm00001d051163 (chromosome 4), Zm00001d051161 (chromosome 4), Zm00001d017279 (chromosome 5), Zm00001d017276 (chromosome 5), Zm00001d017275 (chromosome 5); AtPAL1 (AT2G37040), AtPAL2 (AT3G53260), AtPAL3 (AT5G04230), AtPAL4 (AT3G10340); Os02g0626600, Os02g0626100, Os02g0626400, Os12g0520200, Os02g0627100, Os04g0518100, Os04g0518400, Os05g0427400, Os05g0558900, Os08g0308300, Os12g0461050, Os12g0461300; GLYMA_02G309300, GLYMA_03G181600, GLYMA_03G181700, GLYMA_10G058200, GLYMA_10G209800, GLYMA_13G145000, GLYMA_19G182300, GLYMA_20G180800; HORVU1Hr1G022060, HORVU2Hr1G038120, HORVU2Hr1G038140, HORVU2Hr1G089440, HORVU2Hr1G089540, HORVU6Hr1G058820, HORVU6Hr1G058840, HORVU0Hr1G016330, HORVU3Hr1G005330; BdPAL1 (XM003575348), BdPAL2 (XM003575352), BdPAL3-1 (XM003575190), BdPAL3-2 (XM003575192), BdPAL3-3 (XM003575355), BdPAL3–4 (XM003575356), BdPAL3–5 (XM003575317), BdPAL6 (XM003580096). The distance scale bar indicated amino acid differences per unit length, and the black square indicated the ZmPAL.
