# Supplementary figures and images for: Maize phenylalanine ammonia‐lyases contribute to resistance to Sugarcane mosaic virus infection, most likely through positive regulation of salicylic acid accumulation
Source: Mol Plant Pathol. 2019 Sep 5;20(10):1365–78. doi: 10.1111/mpp.12817 (PMC6792131; doi:10.1111/mpp.12817)

9 dpi

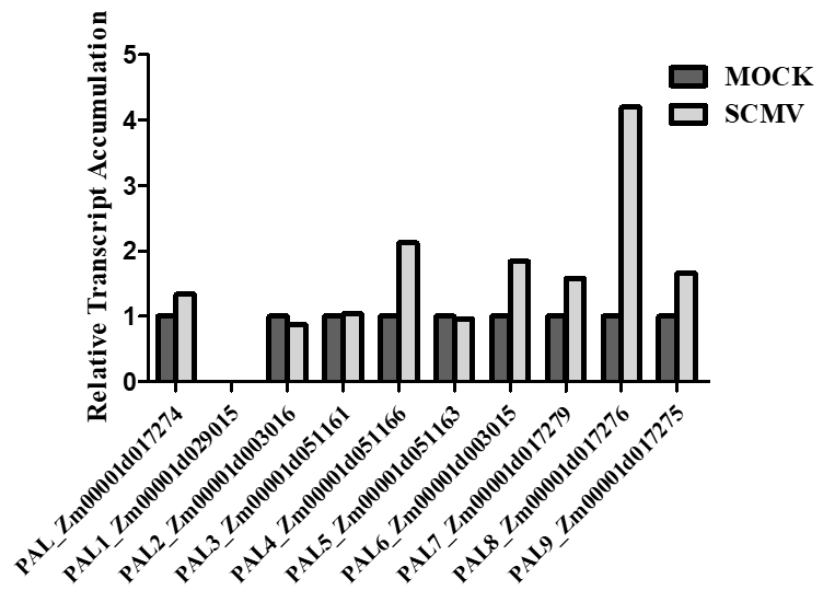

**Fig.S7** RNA-Seq analysis identified *ZmPAL* transcripts that are induced by SCMV infection.

Supplement: Supplementary file 7 — Fig. S7 RNA Seq analysis identified ZmPALs transcripts that are induced by SCMV infection. [file MPP-20-1365-s007.pdf]
