# Supplementary material for: Maize phenylalanine ammonia‐lyases contribute to resistance to Sugarcane mosaic virus infection, most likely through positive regulation of salicylic acid accumulation
Source: Mol Plant Pathol. 2019 Sep 5;20(10):1365–78. doi: 10.1111/mpp.12817 (PMC6792131; doi:10.1111/mpp.12817)
Supplement: Supplementary file 8 — Table S1 Primers used in this study. [file MPP-20-1365-s008.pdf]

Supplementary information: table S1

Table S1 List of primers used in this study.

Name	Sequence
ZmUbi-qRT-F	GGAAAAACCATAACCCTGGA
ZmUbi-qRT-R	ATATGGAGAGAGGGCACCAG
SCMV-CP-qRT-F	GGCGAGACTCAGGAGAATACA
SCMV-CP-qRT-R	ACACGCTACACCAGAAGACACT
ZmPR1-qRT-F	GGCGAGAGCCCCTACTAGAC
ZmPR1-qRT-R	AAATCGCCTGCATGGTTTTA
ZmPR5-qRT-F	GTCATCGACGGCTACAACCT
ZmPR5-qRT-R	CACGGGCAGAAGGTGACT
ZmPAL-silencing-F	ATCCTAGGACAACGCCCGCCT
ZmPAL-silencing-R	TAGCCATGGCGATCTCGGTGCC
XSD198	CTTGTGTTGCTGAGAAAC
XSD199	TCTTGTAAGAGGTCTGC
Oligod(T)	TTTTTTTTTTTTTTTTTT
ZmPAL-qRT- Zm00001d017274-F	AAGGAGAAGAGGAGGGAGGG
ZmPAL-qRT- Zm00001d017274-R	GAAGAAAGAGCAACGCCACA
ZmPAL-qRT- Zm00001d003015-F	GGGAGATGAAGTGAAGTGAAGACA
ZmPAL-qRT- Zm00001d003015-R	GACATAAAACAAAGGAACAGTGAGC
ZmPAL-qRT- Zm00001d017279-F	AGACGGCAGCAAGGTCAACG
ZmPAL-qRT- Zm00001d017279-R	AGGAGGGTCTTGCGATGGTG
ZmPAL-qRT- Zm00001d017276-F	TGGCCCACAGATTGAGGTTA
ZmPAL-qRT- Zm00001d017276-R	AAGCCGTTGTTGTAGTAGTCGTT
ZmCAD-qRT-F	GCCGACTCGCTGGACTACATCA
ZmCAD-qRT-F	TCTCGTCGATGCTGCCGATGAA
ZmCCR-qRT-F	GCGGAAGCAGCCGTACAAGT
ZmCCR-qRT-F	CCTGGAGGTTCTTCACCGTGTC
ZmCOMT-qRT-F	ATGGCAAGGTCATCGTCGTCG
ZmCOMT-qRT-F	AGGCGTTGGCGTAAATGTAGGT
ZmC4H-qRT-F	TGGAGGAGGAGAAGTCCGTGGA
ZmC4H-qRT-F	GCTTGGCGATCTGGTTGCTGAA
